# Single Amino Acid Repeats in the Proteome World: Structural, Functional, and Evolutionary Insights

**DOI:** 10.1371/journal.pone.0166854

**Published:** 2016-11-28

**Authors:** Amitha Sampath Kumar, Divya Tej Sowpati, Rakesh K. Mishra

**Affiliations:** Centre for Cellular and Molecular Biology, Council of Scientific and Industrial Research, Uppal Road, Hyderabad, 500007, India; Russian Academy of Medical Sciences, RUSSIAN FEDERATION

## Abstract

Microsatellites or simple sequence repeats (SSR) are abundant, highly diverse stretches of short DNA repeats present in all genomes. Tandem mono/tri/hexanucleotide repeats in the coding regions contribute to single amino acids repeats (SAARs) in the proteome. While SSRs in the coding region always result in amino acid repeats, a majority of SAARs arise due to a combination of various codons representing the same amino acid and not as a consequence of SSR events. Certain amino acids are abundant in repeat regions indicating a positive selection pressure behind the accumulation of SAARs. By analysing 22 proteomes including the human proteome, we explored the functional and structural relationship of amino acid repeats in an evolutionary context. Only ~15% of repeats are present in any known functional domain, while ~74% of repeats are present in the disordered regions, suggesting that SAARs add to the functionality of proteins by providing flexibility, stability and act as linker elements between domains. Comparison of SAAR containing proteins across species reveals that while shorter repeats are conserved among orthologs, proteins with longer repeats, >15 amino acids, are unique to the respective organism. Lysine repeats are well conserved among orthologs with respect to their length and number of occurrences in a protein. Other amino acids such as glutamic acid, proline, serine and alanine repeats are generally conserved among the orthologs with varying repeat lengths. These findings suggest that SAARs have accumulated in the proteome under positive selection pressure and that they provide flexibility for optimal folding of functional/structural domains of proteins. The insights gained from our observations can help in effective designing and engineering of proteins with novel features.

## Introduction

The human genome encompasses a large number of repetitive elements that constitute more than 50% of the genome [1]. Simple sequence repeats (SSRs) are found in many organisms though they are predominant in eukaryotes than in prokaryotes [2]. They are dynamic elements occurring in diverse patterns and locations in the genome and are almost unique across organisms. SSRs constitute 3% of the human genome. They are considered as the “turning knobs” of evolution that offer genetic diversity as structural and functional elements [3–9]. SSRs evolve faster than point mutations in terms of dynamic genetic variability and there is a bias for the elongation of the repeats rather than their shortening [10]. This points towards functional utility and positive selection of these elements [11, 12]. Among SSRs, tri/hexanucleotide repeats are predominantly present in exons and contribute to single amino acid repeats (SAARs) at the protein level [13].

In addition to being a direct consequence of SSRs in coding regions, SAARs can also be coded by a combination of codons representing the same amino acid. Since proteins have diverse functions, so are the likely roles of SAARs. Some of these repeats are tolerated by the proteins while certain others lead to a monogenic disease state. Abnormal SAAR expansion is known to cause many neurodegenerative diseases in humans, with varying severity and inheritability [14–16]. The role of SAARs in most proteins, however, is not yet understood. It has been shown that SAARs may serve as spacer elements, separating functional domains within the protein [17]. Certain SAARs also regulate transcription [18–20] and facilitate protein-protein interactions [21]. The genes that code for proteins with polyalanine stretches show high selectivity for its base composition and are mostly genes of DNA and RNA binding proteins [22]. While some studies suggest that SAARs are present to merely increase the size of the protein [2], a study has shown that the SAARs in proteins are essential for proper envelope targeting in chloroplasts [23]. However, a general understanding of the functional importance and distribution of SAARs remains to be established.

Previous studies have addressed the distribution of SAARs but many questions regarding their function and evolution remain unanswered [20, 24–27]. In this study, we have used *in silico* analysis to study 22 proteomes of different species of the animal kingdom to explore the evolutionary, structural and functional relevance of SAARs. This study provides clues that may be useful in understanding the organisation and stability of multiple domains in proteins and help in designing proteins with novel structures and functional features.

## Materials and Methods

### Proteome datasets

All proteomes of various vertebrates and invertebrates analysed in this study are listed in Table 1. The proteome datasets were downloaded from the UniProt database [28]. We considered both reviewed and unreviewed sets of proteins for our analysis. In case more than one isoform of a protein was available, we retained only the largest isoform to eliminate redundancy. *Homo sapiens* is the primary organism in our study, and most comparisons were done with respect to the human proteome.

**Table 1:** Invertebrate and vertebrate species under study.

	Class	Species	Common name	Proteome size (million residues)	No of proteins
Vertebrates	Mammalia	*Homo sapiens*	Human	23.35	69025
		*Mus musculus*	Mouse	19.61	43401
	Aves	*Anas platyrhynchos*	Duck	8.14	16374
		*Gallus gallus*	Chicken	9.82	17620
	Pisces	*Danio rerio*	Zebra fish	20.55	40895
		*Oryzias latipes*	Japanese rice fish	11.97	24633
	Reptitilia	*Ophiophagus Hannah*	King cobra	7.68	18387
		*Anolis carolinensis*	Green anole	10.13	19109
	Amphibia	*Xenopus tropicalis*	Frog	12.59	23486
Invertebrates	Protozoa	*Leishmania infantum*	Leishmania	5.12	8045
		*Trypanosoma cruzi*	Trypanosoma	5.11	10806
	Porifera	*Amphimedon queenslandica*	Sponge	11.68	29741
	Cnidaria	*Nematostella vectensis*	Starlet sea anemone	8.26	24435
	Platyhelminthes	*Echinococcus multilocularis*	Fox tapeworm	5.22	10333
		*Clonorchis sinensis*	Chinese liver fluke	7.22	13606
	Nemathelminthes	*Caenorhabditis brenneri*	Nematode worm	11.99	29982
		*Pristionchus pacificus*	Parasitic nematode	8.41	29076
	Annelids	*Helobdella robusta*	Californian leech	8.77	23328
	Arthropoda	*Drosophila melanogaster*	Fruit fly	12.71	19447
		*Tetranychus urticae*	Two spotted spider mite	6.51	18082
	Mollusca	*Crassostrea gigas*	Pacific oyster	11.65	25982
	Echinodermata	*Strongylcentrotus purpuratus*	Purple sea urchin	14.26	28567

### Identification of SAARs

A homopolymer of any amino acid with a length of > = 5 was considered an SAAR. Every occurrence of an uninterrupted SAAR is considered as an event. A protein can thus have more than one event. For each protein in the proteome, protein id, the number of events, amino acids involved in the events, and the repeat length of each event were recorded. To identify and categorize SAARs in the proteomes, a custom Perl script was used.

We calculated SAAR density and total density for all the amino acids to discriminate their presence as repeats vs. their total occurrence. SAAR density is calculated as the number of amino acid residues present as repeats by the total proteome size normalized to one million residues. Similarly, total density is calculated as the total number of residues of any particular amino acid by the total proteome size normalized to one million residues.

### Functional and structural annotation

For functional annotation of our proteome datasets, we used the PfamA [29] domain database that provides reviewed domain annotation. D2P2.pro [30] was used for disordered domain annotation. Secondary structure information was annotated using the UniProt database for the human proteome [28]. We also populated the secondary structure annotation for all the proteins with SAARs from the PDB database where complete solved structures were available [31].

### Protein orthologs and SAAR conservation

To identify protein ortholog pairs, we used an all vs. all blastp search between human and other proteomes. The blastp hits (human vs. each of the other organisms under study) with highest identity scores for each protein were searched for the conservation of functional domains. If the domains were conserved, the pairs were considered orthologous and were included in the study. Orthologs were grouped into two categories based on the level of repeat conservation. In the first category, we considered ortholog pairs where the order of SAAR events and their lengths are conserved. The second group included the orthologs where the SAAR events are conserved but not the lengths.

### Gene ontology

Gene ontology (GO) annotations were done using the Panther database [32]. Statistical overrepresentation test for molecular function GO terms was performed with proteins containing SAARs, using the human proteome as background. Only those categories that showed a p-value of <0.05 were retained.

### Codon organisation of Single amino acid repeats in the Human genome

To understand the base composition of protein repeats, we analysed the DNA sequence corresponding to each SAAR from the coding region of the human genome hg38. The CDS annotation was retrieved from the UCSC table browser [33]. We retrieved the codon fraction information for the Human CDS using the GenScript Codon Usage Frequency Table Tool [34]. The algorithm implemented for the extraction of genomic regions contributing to SAARs ensured maximum inclusion of such regions. For overlapping regions of CDS, the transcript with the highest number of repeat events was taken in case of common SAAR events in both the transcripts, else all unique repeat associated regions were considered. A minimum of 12 nucleotide-trimer repeats (4 repeating units) in the coding region was considered as one SSR event.

### Statistical analysis

For categorization of the relationship between amino acids based on their SAAR density vs. the total density in the proteome, we performed hierarchical clustering on Canberra distance of amino acids. Canberra distance is a measure to compute the distance between paired points in a vector space. For two vectors in an n-dimensional vector space, Canberra distance *d* can be calculated as:

$$d\left(\text{p},\text{q}\right)=\sum_{i=1}^{n}\frac{\left|p_{i}-\;q_{i}\right|}{\left|p_{i}\right|+\;\left|q_{i}\right|}$$

where p and q are two vectors of real numbers.

The Canberra distance of amino acids was computed in R. The SAAR densities and total densities of all amino acids were stored in two vectors such that the nth element of both vectors correspond to the same amino acid. Both the vectors were fed into R’s dist function to calculate the Canberra distance.

Hierarchical clustering was performed using the hclust function in R. Linear regression analysis was performed using lm function of R, and the results were plotted using ggplot2 package[35]. Fisher’s exact test and Chi-squared tests were performed using GraphPad [36].

### Repeat expansion diseases

We looked for the repeat conservation in genes associated with repeat expansion diseases [34]. Orthologous genes for these human genes were searched using the Homologene database for various animal and plant genomes, some of them including *Pan troglodytes*, *Canis lupus familiaris*, *Bos taurus*, *Mus musculus*, *Rattus norvegicus*, *Oryza sativa*, *Arabidopsis thaliana*, *Caenorhabditis elegans*, *Anopheles gambiae str*. *PEST and Drosophila melanogaster*. We studied repeat conservation in genes associated with the diseases Huntingtin (HTT), oculopharyngeal muscular dystrophy (PABPN1), and spiro-cerebellar ataxia type 3 (ATXN3) genes.

## Results

### 1. SAARs in the Human proteome

#### 1.1 Distribution of SAARs in human proteome

**Fig 1:**
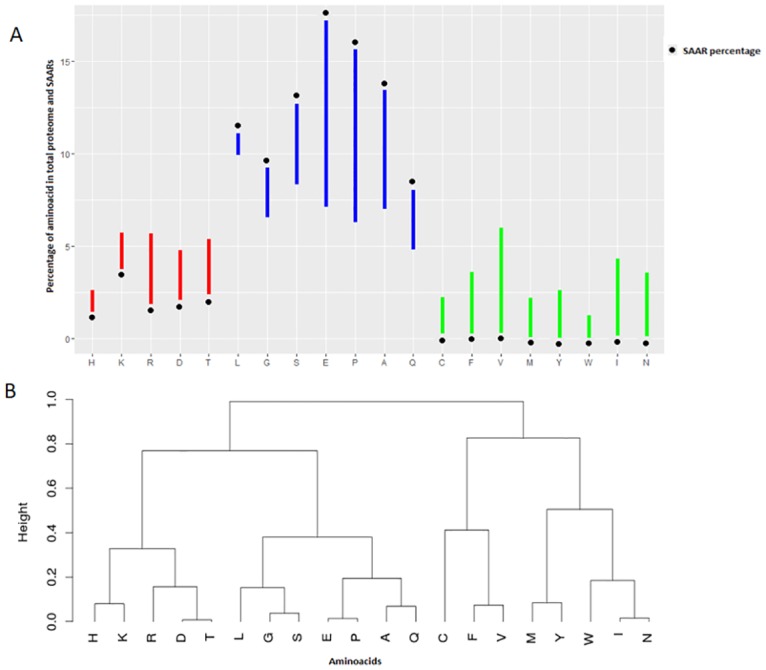
Comparison of amino acid and SAAR density in the human proteome. The amino acid density and SAAR density normalized to 1 million residues were calculated for all the 20 amino acids. (A) The percentage of each amino acid in the whole proteome and SAARs are represented as vertical bars. The black dot represents the SAAR percentage in each bar and the opposite end indicates amino acid percentage in the whole proteome. The bars are grouped by colour to indicate the three distinct patterns observed (see text) (blue—group1, red—group2, green—group3) (B) A distance-based dendrogram was plotted for the values of amino acid density and SAAR density for all the 20 amino acids. A distinct pattern of preference for SAARs vs. proteome density is seen clustered as three groups as described in (A) (see text)

SAARs with a minimum length of 5 residues were searched for in the human proteome using Perl scripts developed in-house. About ~14% of the proteins in the human proteome contain SAARs. A total of 11852 SAAR events were found in 9780 proteins, indicating that a significant proportion of the proteins have >1 event. We asked if SAARs are random events linearly correlated to the total density of an amino acid. To answer this, we calculated the total density—abundance of a given amino acid in a proteome normalized to million residues, and SAAR density—the abundance of a given amino acid as part of repeats normalized to proteome size, for all amino acids (see methods). Compared to the total density of each amino acid, SAAR density showed a varying abundance (Fig 1A), indicating selective enrichment of SAARs. Using hierarchical clustering of mean Canberra distance for each amino acid (see methods), amino acids could be categorized into three distinct groups based on the ratio of their density in whole proteome to their SAAR density (Fig 1B)—First group (glutamic acid, proline, alanine, serine, leucine, glycine and glutamine) consists of those amino acids which are abundant in the proteome and also show high SAAR densities. Glutamine, proline, alanine and glutamic acid show particularly high abundance of repeats. The second grouping is of lysine, threonine, aspartic acid, arginine and histidine, which show a relatively higher total density but much lesser SAAR density. In the last group, we see a set of amino acids that are intolerant to repeats and are also less dense in the whole proteome. This group includes cysteine, phenylalanine, valine, methionine, tyrosine, tryptophan, isoleucine and asparagine. Within the last group, valine, isoleucine and asparagine show a slightly higher density in the whole proteome but remain intolerant to repeats.

#### 1.2 Physical properties of amino acids in SAARs

**Fig 2:**
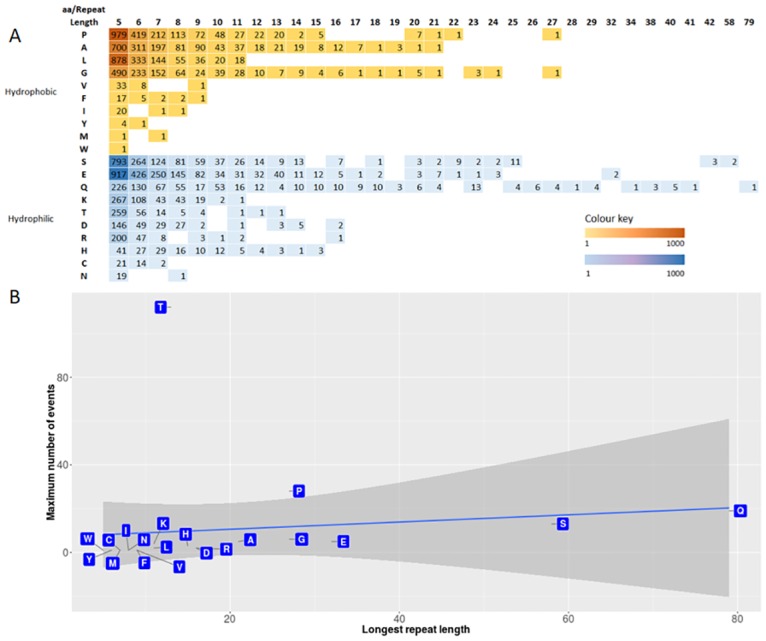
SAAR repeats lengths and occurrences in the human proteome. A) For each amino acid, the number of SAAR events were calculated with a minimum length of 5 residues. The heat map shows the number of events in the various repeat lengths, with each cell indicating the number of events grouped by their physical properties (Hydrophobic—orange, Hydrophilic—blue). Empty cells indicate 0 events. B) For all the amino acids in the human proteome, the longest repeat present and the maximum number of SAAR events present within a protein was calculated. A scatter plot shows the longest repeat length (x-axis) vs. the maximum number of events tolerated in a single protein (y-axis), the grey shaded region indicates 95% confidence in a linear regression analysis.

Proteins with stretches of hydrophobic amino acids often fold in a way such that the hydrophobic regions are shielded from solvent access [36]. We therefore asked whether amino acids with polar or non-polar side chains show any trend related to their number or length of SAAR events. To address this question, we grouped the SAARs based on whether the amino acid is hydrophobic or not. In both categories, we analysed the number of SAAR events and the SAAR density (SAAR events per million amino acid residues), the maximum number of events tolerated by a single protein, and the longest repeat in the whole proteome, for every amino acid. The hydrophobicity of an amino acid does not appear to be a factor for the abundance or length of its SAAR events (Fig 2A). However, we observed a relation between the abundance and length of SAAR events; amino acids that show a higher abundance of SAARs are also likely to be tolerated as long repeats. To test the validity of this observation, we compared the maximum number of SAAR events that could be accommodated within a single protein and longest SAAR length for all the amino acids. A linear regression fit at 95% confidence indicated that most amino acids that contribute to a high number of repeat events in a single protein can also be tolerated as long SAARs (Fig 2B). Exceptions to this are threonine and proline (p < 0.05, linear regression), which exhibit a tendency to be present as shorter but abundant events within a protein. For example, the human protein Mucin-2 (Q02817), which coats the epithelia of many mucus membrane-containing organs preventing bacteria from entering the inner mucus layer[37], has 112 threonine SAAR events, the most in all human proteins.

#### 1.3 Functional and structural association of SAAR in the protein

We next asked if we could classify the proteins containing SAARs based on their function. To study this, we annotated all human proteins containing SAARs using the molecular function gene ontology terms from the Panther database and further grouped these proteins based on the hydrophobicity of their SAARs (S1 Fig). Using statistical over- and underrepresentation tests (see Methods), we observed a preference of SAARs to specific functions; proteins containing hydrophobic SAARs showed enrichment for receptor activity whereas those containing hydrophilic SAARs were underrepresented for the same. In general, proteins containing SAARs were overrepresented in various binding activities, particularly chromatin binding, mRNA binding, transcription factor binding, cytoskeletal protein binding, DNA, and RNA binding.

**Fig 3:**
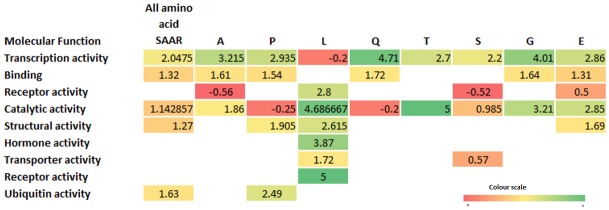
Distribution of various molecular activities associated with SAAR containing proteins in the human proteome. Molecular function class for proteins was annotated using the Panther database. Proteins with any amino acid repeat and proteins with a particular abundant amino acid repeat such as proline, alanine, leucine, glutamine, threonine, serine, glycine and glutamic acid are reported. Each cell in the heat map shows the fold enrichment between expected and observed frequency in reference to the human proteome. The colours in the heatmap scale from red to green where the fold enrichment is from 0 to 5 respectively.

A closer observation of the molecular functions among proteins containing SAARs of specific amino acids revealed high enrichment and clustering to certain functions (Fig 3). Most of the amino acid repeats like alanine, proline, glutamine and glycine are ~1.5 fold enriched for various binding activity. It is also seen that transcription activity associated proteins are enriched for glutamine and glycine SAARs. We also see a high enrichment in proteins with lysine and threonine SAARs for catalytic activity.

Most proteins have known motifs related to distinct functions. To understand the contribution of SAARs towards functional motifs in proteins, we annotated our proteome datasets for functional domains using the Pfam-A database. We grouped the SAARs present within or outside the functional domains. Very few SAARs (1666 out of 11842, ~15%) were present in the functionally annotated domains while most of the SAARs mapped outside any known functional domain (S1 File). This suggests that SAARs are generally not part of any known functional domains and do not have any distinct functional features on their own.

**Fig 4:**
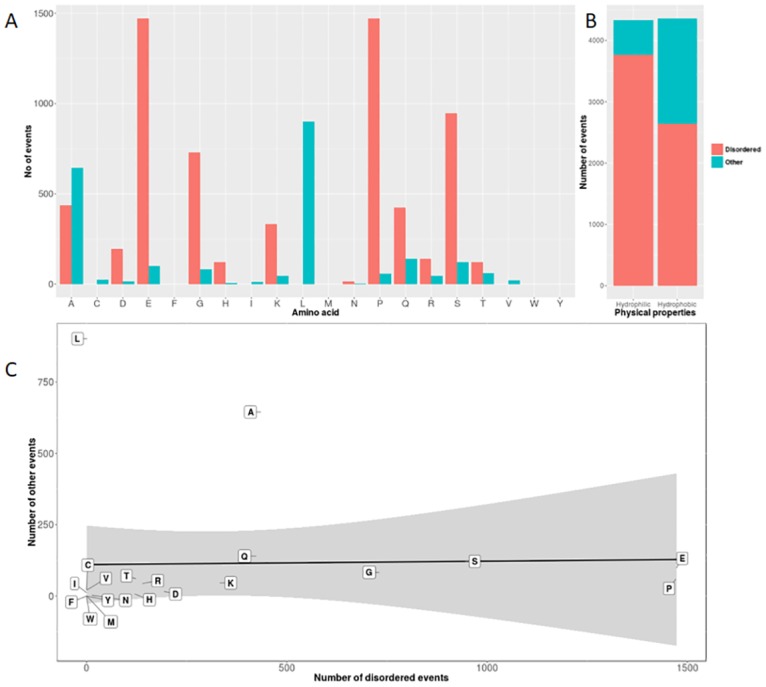
Disordered and regular domains in the human proteom. (A) SAARs were categorized based on their presence in the regular or disordered region of the protein, annotated using the D2P2.pro database. The X-axis denotes each of the 20 different amino acids and the Y-axis shows the number of SAAR events in the disordered and regular part of proteins in the human proteome. (B) A stacked bar plot shows the number of events in the disordered region of the protein (red) and the regular region (blue) categorized based on the amino acids’ physical properties. (C) Scatter plot with linear regression analysis for each amino acid showing the number of SAAR events in the disordered regions (X-axis) vs. SAAR events in the other regions (Y-axis). Grey shaded area indicates 95% confidence interval.

Proteins have a complex structure determined by the three-dimensional folding of different structural domains, while some regions within the proteins remain unfolded and disordered. Disordered domains offer an advantage to the protein by making it more structurally flexible and facilitate optional folding [38]. To infer any association of SAARs with such regions, we used the D2P2.pro database to annotate disordered regions in our SAAR containing protein datasets. We could annotate 8689 out of the total 11852 SAARs found. Interestingly, a large number of annotated events (6406 out of 8689, 73.7%) were part of disordered regions. We further classified the events for each individual amino acid to assess the preference of various amino acids to be part of disordered regions. Repeats of many amino acids displayed a preference for occurrence within disordered domains. Particularly, more than 90% of the SAARs involving the amino acids proline, serine, glycine, glutamic acid and aspartic acid were in disordered regions (Fig 4A). This was further confirmed using linear regression analysis, which showed that the fit for most amino acids is inclined towards disordered regions (Fig 4C). Contrarily, leucine and alanine showed an opposite preference, with a majority of their SAARs falling in non-disordered regions. When we grouped the SAARs based on the hydrophobicity of amino acids, we observed that hydrophilic amino acid repeats are more inclined to be part of disordered domains (p < 0.0001, Fisher’s exact test, Fig 4B). This observation points towards the role of SAARs in providing flexibility to the proteins rather than being part of functional domains.

#### 1.4 Codon structure of Single amino acid repeats in the Human genome

SAARs can be a direct consequence of simple sequence repeats (SSRs) present in coding sequences or a combination of codons coding for the same amino acid. Using coding sequences corresponding to SAARs in the human genome hg19, we set out to identify the fraction of SAARs contributed by SSRs. For all the amino acids that are present as SAARs, we calculated the number of times they were present as SSRs (a tandem triplet repeat of at least 12 nucleotides (4 codons)) (S2 Fig). We observed that 32% of SAAR events were present as SSRs at the genomic level and the rest were constituted of a combination of codons. Interestingly, just 46 SAAR events were split across exons at the genomic level (data not shown).

We further asked if there was a bias in codon usage at sequences contributing to SAARs. To check this, we gathered the codon usage for the entire human CDS using GenScript Codon Usage Frequency Table and compared it with the codon fraction for SAARs. For most amino acids, SAARs comprising of mixed codons showed no significant bias compared to codon usage of human CDS (p > 0.01, Chi-squared test, Table 2). However, SAARs arising from SSRs at the genomic level showed a strong preference to certain codons. The most drastic example is of threonine repeats; >99% (1393 out of 1404) codons encoding threonine repeats were ACC repeats whereas ACC accounts for only 36% of all threonine amino acids in the human proteome. Other amino acids that display this bias are glutamine (99% CAG), histidine (96% CAC), leucine (93% CTG), lysine (92% AAG), glutamic acid (88% GAG), aspartic acid (88% GAT) and glycine (84% GGC). The bias shown by these amino acids was statistically significant (p < 0.0001, Chi-squared test, Table 2).

**Table 2:** Codon fraction for SAAR coding regions against codon fraction of CDS in the human genome.

Codon	Amino acid	Codon fraction (entire human CDS)	SAAR codon fraction (Mixed)			SAAR codon fraction (SSR)		
			Fraction	No of codons	p value (Chi-squared test)	Fraction	No of codons	p value (Chi-squared test)
GCA	Alanine	0.23	0.21	1372	7.7466E-06	0.09	222	1.5995E-17
GCC	Alanine	0.4	0.32	2085		0.41	1029	
GCG	Alanine	0.11	0.27	1723		0.38	958	
GCT	Alanine	0.26	0.2	1291		0.13	326	
TGC	Cysteine	0.55	0.55	17	1	0.88	68	3.28376E-11
TGT	Cysteine	0.45	0.45	14		0.12	9	
GAC	Aspartic acid	0.54	0.45	151	0.07095	0.12	55	3.5461E-17
GAT	Aspartic acid	0.46	0.55	187		0.88	396	
GAA	Glutamic acid	0.42	0.46	3166	0.41768	0.12	380	1.21458E-09
GAG	Glutamic acid	0.58	0.54	3655		0.88	2682	
TTC	Phenylalanine	0.55	0.57	24	0.68767	0.1	12	1.492E-19
TTT	Phenylalanine	0.45	0.43	18		0.9	110	
GGA	Glycine	0.25	0.19	880	0.08689	0.1	180	3.03723E-24
GGC	Glycine	0.34	0.41	1925		0.84	1629	
GGG	Glycine	0.25	0.19	892		0	0	
GGT	Glycine	0.16	0.22	1033		0.07	129	
CAC	Histidine	0.59	0.59	245	1	0.96	489	5.3585E-14
CAT	Histidine	0.41	0.41	171		0.04	20	
ATA	Isoleucine	0.16	0.1	4	0.24935	0	0	5.03104E-09
ATC	Isoleucine	0.48	0.5	20		0.77	24	
ATT	Isoleucine	0.36	0.4	16		0.23	7	
AAA	Lysine	0.42	0.41	663	0.83943	0.08	26	5.62857E-12
AAG	Lysine	0.58	0.59	944		0.92	290	
CTA	Leucine	0.07	0.06	174	0.20262	0	0	4.80722E-23
CTC	Leucine	0.2	0.24	737		0.07	86	
CTG	Leucine	0.41	0.49	1528		0.93	1071	
CTT	Leucine	0.13	0.09	294		0	0	
TTA	Leucine	0.07	0.03	80		0	0	
TTG	Leucine	0.13	0.09	291		0	0	
ATG	Methionine	1	0	0	NA	1	12	NA
AAC	Aspargine	0.54	0.54	27	1	1	25	2.7169E-20
AAT	Aspargine	0.46	0.46	23		0	0	
CCA	Proline	0.27	0.27	4148	0.18835	0.15	253	3.13854E-46
CCC	Proline	0.33	0.25	3771		0	0	
CCG	Proline	0.11	0.16	2397		0.55	912	
CCT	Proline	0.28	0.32	4953		0.3	499	
CAA	Glutamine	0.25	0.44	1362	1.1447E-05	0.01	42	2.98077E-08
CAG	Glutamine	0.75	0.56	1705		0.99	3042	
AGA	Arginine	0.2	0.08	60	0.01284	0.03	4	4.53841E-15
AGG	Arginine	0.2	0.23	173		0.16	21	
CGA	Arginine	0.11	0.1	72		0	0	
CGC	Arginine	0.19	0.22	163		0.37	48	
CGG	Arginine	0.21	0.32	236		0.44	57	
CGT	Arginine	0.08	0.06	44		0	0	
AGC	Serine	0.24	0.24	1321	0.41072	0.64	1061	1.08417E-22
AGT	Serine	0.15	0.11	582		0	4	
TCA	Serine	0.15	0.11	588		0.02	33	
TCC	Serine	0.22	0.3	1063		0.3	506	
TCG	Serine	0.06	0.06	330		0.02	32	
TCT	Serine	0.18	0.18	999		0.02	25	
ACA	Threonine	0.28	0.11	240	1.969E-06	0.01	11	4.08504E-37
ACC	Threonine	0.36	0.47	1061		0.99	1393	
ACG	Threonine	0.12	0.25	568		0	0	
ACT	Threonine	0.24	0.18	407		0	4	
GTA	Valine	0.11	0.08	7	0.239689503	0	0	2.78621E-24
GTC	Valine	0.24	0.19	18		0	0	
GTG	Valine	0.47	0.57	53		1	29	
GTT	Valine	0.18	0.16	15		0	0	
TGG	Tryptophan	1	0	0	NA	1	4	NA
TAC	Tyrosine	0.57	0.2	2	1.67448E-09	0	0	1.52397E-23
TAT	Tyrosine	0.43	0.2	8		0	0	

Cells highlighted in green colour show very significant difference (p < 0.0001, Chi-squared test) compared to fraction of entire human CDS.

### 2. Comparative analysis of SAARs among vertebrates and invertebrates

#### 2.1 Distribution of SAARs in various species

**Fig 5:**
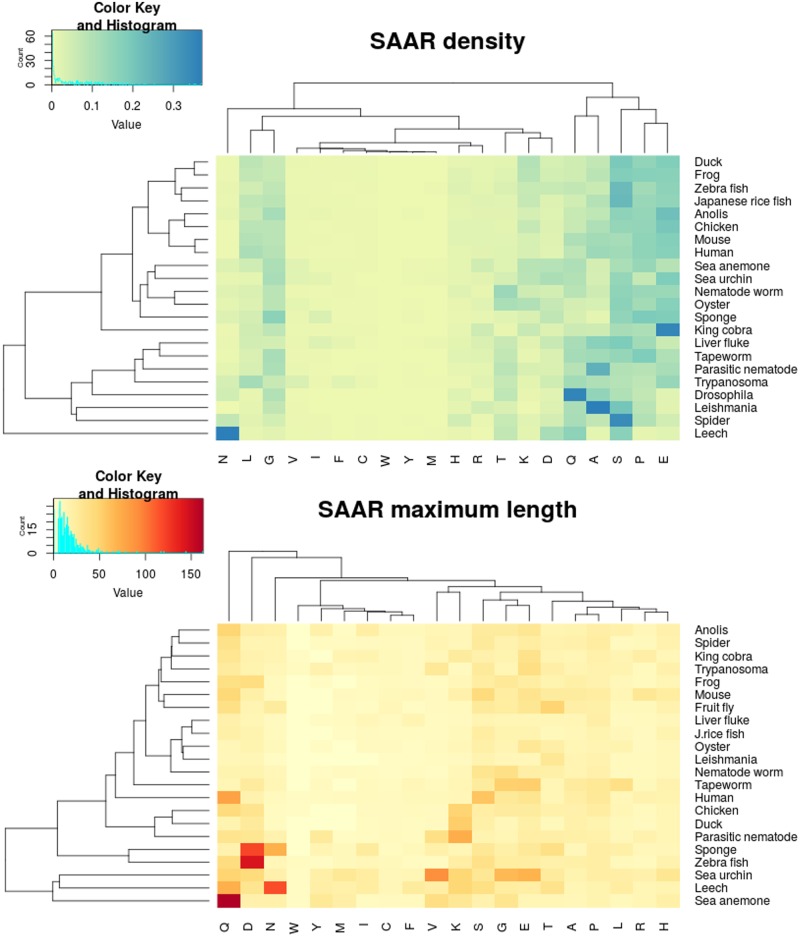
SAAR density and longest SAAR among all proteomes. (A) SAAR density was calculated and normalized to one million residues for the indicated proteomes and plotted as a heatmap where the X-axes show individual amino acid associated repeats and Y-axes have all the organisms under study. The plot is two way clustered to group amino acids and species with similar densities. (B) A heat map was generated for the longest repeat length for all the amino acids in each of the vertebrate and invertebrate proteomes under study. The plot is two-way clustered between the longest SAARs (X-axis) and the proteomes (Y-axis).

To study the changes in the abundance of SAARs during evolution, we compared the SAAR density per million residues (normalized to proteome size) of all the amino acids in the 21 proteomes studied (Fig 5A). We performed a two-way clustering to group amino acids and species that show similar SAAR densities. The SAAR density is found to be relatively similar among all the species. Alanine, serine, aspartic acid, glutamic acid and glutamine SAARs show a high density across most of the proteomes. Interestingly, certain amino acids show high density in invertebrates compared to vertebrates. Notable examples of this trend include serine SAARs in spider, glutamine SAARs in *Drosophila*, and alanine SAARs in *Leishmania*. Leech showed a very high density of aspargine SAARs, which was not observed in any other species studied. Among the vertebrates studied, the king cobra proteome showed the highest density for glutamic acid SAARs. We did a similar analysis for the number of SAAR events (normalized to the total number of SAARs events in the proteome), where a pattern similar to SAAR density was observed (S3 Fig). The amino acids valine, isoleucine, phenylalanine, cysteine, tryptophan, tyrosine, and methionine are consistently low in all proteomes including humans where these amino acids are clustered in the third group (Fig 1A, green bars). The high density and events of certain SAARs across all the proteomes suggests that they have been selected for and retained during evolution. Since the SAAR density and number of events were equally abundant in all the proteomes studied, we also wanted to look for the longest SAAR tolerated for all the amino acids in these proteomes (Fig 5b). For most amino acids, the longest SAARs were observed in invertebrate proteomes. Sea Urchin has the longest SAAR for valine, glycine, methionine, and glutamic acid. Similarly, sea anemone and leech have the longest glutamine and aspargine repeats respectively. Similarly, SAARs longer than 100 residues were mostly seen in invertebrate proteomes where the sea anemone proteome has a glutamine SAAR of 163 residues, the leech proteome has an SAAR of 117 asparagine residues and the sponge proteome that has an aspartic acid SAAR of 120 residues. Zebrafish was the only vertebrate in the study with an SAAR longer than 100 amino acid residues, with an SAAR of 144 aspartic acid residues.

#### 2.2 Conservation of SAARs of human proteins among orthologs

**Fig 6:**
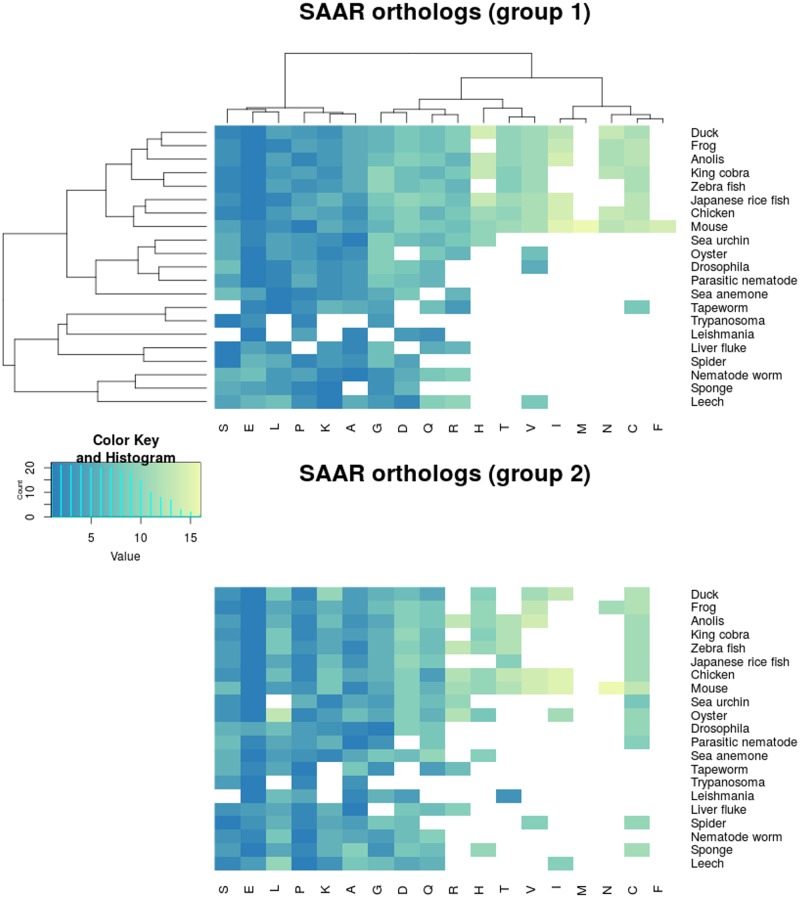
Group 1 and group 2 orthologs. The data shows the SAAR conservation between orthologous proteins of several vertebrate and invertebrate proteomes. Amino acids are ranked by the frequency of conservation (lower value indicates better conservation). Group 1 (top) contains human ortholog pairs in which the SAAR events are conserved in terms of events and repeat lengths. Group 2 (bottom) contains human ortholog pairs that are conserved by the number of events but not their repeat length. Group 1 is two way clustered to group amino acids and species with similar conservation. Group 2 follows the order of amino acids and species of group 1 to allow an easier comparison between plots.

We have shown that the SAARs are equally abundant in terms of their density and frequency among all the 22 proteomes studied. To understand the conservation of repeats among various species, we looked at ortholog pairs of human proteins. We considered two parameters to define orthologous pairs of proteins—a minimum of 35% sequence identity and matching domains/domain families as annotated by the Pfam-A database. For all the orthologous pairs, we looked for the conservation of the repeats and categorized them into two groups based on the degree of repeat conservation. The first group looked for a stringent match of SAAR length and the order of conservation (in cases where more than one event was present within a protein). Most of the conserved SAAR events in the first group were of short lengths (~5–9 aa residues) (S4 Fig). The second group included those protein pairs where the SAAR events were conserved in the same order but their lengths were different. For both the groups, we ranked each amino acid based on the number of conserved human ortholog pairs. These ranks were then plotted as a heatmap (Fig 6). A good conservation was observed for SAARs containing alanine, leucine, proline, glycine, serine, lysine and glutamic acid in both the groups. Glutamine SAARs were moderately conserved in both groups. The second group exhibits good conservation with alanine, serine, and glycine associated SAARs. Threonine, cysteine, and asparagine SAARs were seen to be moderately conserved, predominantly among vertebrates.

#### 2.3 Gain of SAAR events among orthologous proteins along the evolutionary scale

In our analysis, we identified several proteins which showed a large number of SAAR events. For example, the human Formin2 protein, which is responsible for actin cytoskeleton organisation and cell polarity, has 28 events of proline SAAR of length 5–6 residues along with one glycine and two glutamine repeat events [39]. To understand if the high number of SAAR events were acquired progressively during evolution or were gained by a common ancestor early on, we compared several human proteins with high SAAR events with their orthologs across species. Orthologs of Formin2 show a clear decline of proline SAAR events as we go down the taxonomic hierarchy. We observed this trend in a few other candidate proteins as well, irrespective of the amino acid repeat they contain (Table 3). These examples suggest that the acquisition of many SAAR events by a protein is not spontaneous, and instead is a consequence of several events of positive selection for amino acid repeats.

**Table 3:** Protein orthologs for SAAR containing proteins among animals (one event is a mono aminoacid stretch of ≥5 residues).

Organism	FMN2 (Proline repeat events)	MUC2 (Threonine repeat events)	KMT2D (Glutamine repeat events)	MUC5B (Threonine repeat events)
Human	28	112	19	12
Chimpanzee	15	0	20	3
Mouse	14	3	18	0
Chicken	11			0
Lizard	12	15	17	9
Tropical clawed frog	9	14	6	0
Zebrafish	6	4	1	1
Fruit fly	6	0	0	-

### 3. Secondary structures of SAARs in proteins with solved structures

To study the structural confirmation of SAARs, we annotated the SAAR events with secondary structure information from the UniProt database, which was available for only 58 SAAR events in the human proteome. As the number of SAAR events that could be annotated using UniProt was too low to infer meaningful trends, we instead used the data of all proteins with solved structure information from the PDB database of all organisms. 16731 proteins with solved structures contained at least one SAAR event, out of which less than 1% had secondary structure annotation for SAAR regions. This is in concordance with our observation that most of the SAARs map to disordered regions of the protein, and hence may not be contributing to the secondary structure of the protein directly.

### 4. Repeat expansion diseases

**Fig 7:**
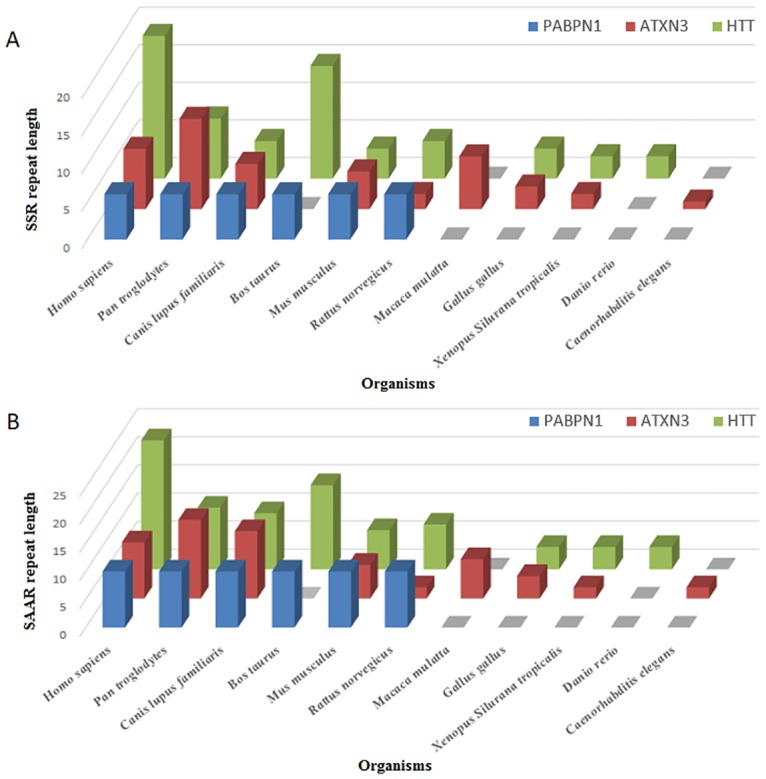
Number of repeats in genes associated with repeat expansion diseases. The genes PABPN1, ATXN3 and HTT are associated with repeat expansion diseases. PABPN1 is associated with PolyA (GCG) expansion that causes OPMD, ATXN3 and HTT cause SCA3 and HTT, respectively upon abnormal expansion of PolyQ (CAG). (A) shows the length of SSR present in the CDS of the genes (B) shows the length of SAARs in the proteins of the respective genes.

Many studies show that certain SAARs, upon abnormal expansion, cause various neurodegenerative diseases in humans [9, 14, 20, 34]. We looked at the orthologs of such proteins and their SAARs. In cases where the severity of the disease is linked to the extent of repeat elongation (for example, CAG expansion diseases), orthologous proteins also had the repeats but at variable lengths, irrespective of the phylogenetic lineage (Fig 7, S1 Table). However, in proteins responsible for PolyA expansion diseases like OPMD, where even a single codon expansion of the GCG repeat (PABPN1 gene) would cause the diseased state, all the orthologous proteins had perfect conservation of the repeat lengths. These observations indicate that not only did repeats get selected during evolution, but they are also maintained to prevent abnormal expansion that can cause diseases.

## Discussion

Repeat elements are the most abundant class of DNA elements in the genomes, particularly in higher eukaryotes. However, repeat regions are less common in proteins. SSRs contribute to amino acid repeats if they occur in exons. Our analysis shows that only 32% of SAARs arise due to SSRs, indicating that SAARs are not merely a consequence of SSRs in the genome but have evolved independent of SSRs. This is further corroborated by the observation that few amino acids (glutamic acid, proline, and alanine) show much higher SAAR density across most proteomes compared to other amino acids. We also noted that some amino acids like glutamic acid, serine and glutamine are tolerated at much higher repeat lengths than others. In many cases, the SAAR frequency of an amino acid is proportional to the lengths that could be tolerated. However, amino acids such as threonine, lysine, arginine and aspartic acid are never found as large repeats albeit having frequencies comparable to glutamine, which has the longest repeat in the human proteome (Fig 2A). These observations indicate that these amino acid repeats are under constant selective pressure to be retained by the proteins but not expanded.

Our study has shown that SAARs are not associated with any functional domains/motifs within the proteins. Instead, they mostly overlap disordered regions of proteins. This is in agreement with the idea that SAARs could act as spacer elements separating two or more functional domains within a protein [17]. Disordered regions are advantageous to proteins as they offer structural flexibility and facilitate optional folding. We hypothesize that proteins acquire SAARs based on the structural flexibility needed for their function. Our molecular function gene ontology analysis shows that proteins containing SAARs are overrepresented in various binding activities such as chromatin binding, DNA and RNA binding, transcription factor activity, etc. By being structurally malleable, these proteins can adapt to bind their targets efficiently. For example, a DNA-binding protein could tolerate imperfect binding sequences, and a transcription factor could adapt itself depending on whether its target promoter is in a highly euchromatic region or not. This hypothesis is further strengthened by amino acid specific categorization of molecular functions; proteins containing proline and glutamic acid repeats, which show the highest tendency to be part of disordered regions, are enriched for chromatin and nucleic acid binding related functions whereas proteins containing leucine repeats, which never occur in disordered regions, are underrepresented for the same. On the other hand, proteins with leucine repeats are overrepresented for functions such as antigen binding and receptor activities, which require precise binding to targets. SAARs of various amino acids, therefore, may function as components that facilitate folding, domain structuring and stability of multi-domain proteins, as also hinted by few previous studies [40–42].

Comparison of SAARs across many proteomes showed that repeats of certain amino acids are equally abundant along the evolutionary tree. Our analysis on repeats of orthologous proteins indicated that SAAR conservation is limited to events of smaller lengths, mostly in the range of 5–9 residues. We also observed in the human proteome that codons encoding repeats are very rarely split across exons (46 out of 11852 events). Furthermore, there are cases like Formin2 and MUC2 genes, where we observe a steady increase in the number of SAAR events in the human proteins as compared to the corresponding homologues in lower organisms. Taken together, these observations suggest that many proteins acquired small SAARs early on during evolution, when exon-intron complexity was minimal. As complexity evolved, proteins acquired more events to augment their adaptability. However, long amino acid repeats are likely to be recent acquisitions. In fact, repeats longer than 15aa are unique to each species, with an exception of three events shared by human and mouse (S4 Fig). However, we did observe that in some cases (~13%), such long SAAR events are replaced in orthologous proteins by another amino acid repeat of similar polarity (data not shown). Hence, we think these are recent and parallel acquisitions, driven by the need of a protein to enhance its functionality.

The functional relevance of SAARs is further substantiated by their roles in causing disease phenotypes. We looked at genes that cause neurological disorders upon expansion of CAG repeats. These genes exhibit a gain of function upon expansion of repeat and the disease severity is proportional to the length of repeat [34]. Orthologs of human genes in plant genomes lack the repeat region while present at varied repeat lengths in the animal genomes. This suggests that animal genomes acquired SAARs for improved functionality with the trade-off that the protein is now vulnerable to a diseased state upon abnormal expansion of the repeat.

## Conclusion

Our analysis shows that SAARs may have been accumulated under selective pressure and, therefore, have functional relevance. Since a great majority of SAARs are part of disordered regions, their function seems indirect, by providing flexibility and stability to proteins in the context of other functional domains. The widespread occurrence of SAARs across the evolutionary landscape, as well as their occurrence in core exonic regions and not at the intron-exon boundaries, indicates that much of SAARs are not a late addition to the proteins but have been essential part of proteins from early on. There is, however, evidence of recent acquisitions/expansions, in particular of the increasing SAAR content in more complex organisms. These findings help us understand the evolutionary determinants that enhance functional features of proteins and can be used in better design of proteins with novel properties.

## Supporting Information

S1 Fig Functional classification grouped by the physical properties of SAARs. Distribution of various functional and molecular activity classes (X-axis) for SAAR associated proteins categorized based on their physical properties is shown. The functional classes were defined from Gene ontology annotations. The plot shows the fold enrichment (Y-axis) between expected and observed frequency in reference to the human proteome. The blue bars and red bars indicate hydrophobic and hydrophilic SAARs, respectively.
(TIF)

S2 Fig SAARs present as SSRs at the genomic level in human. For all the coding regions corresponding to SAARs in the Human proteome, we calculated the number of times they were present as simple sequence repeats (SSRs). The X-axis shows the different amino acids and the Y-axis shows the number of repeat events in the proteome (red bars) and genome (blue bars).
(TIF)

S3 Fig SAAR events in all proteomes. SAAR events were calculated and normalized to one million residues for the indicated proteomes and plotted as a heatmap where the X-axes show individual amino acid associated repeats and Y-axes have all the organisms under study. The order of amino acids and species was kept consistent with that of SAAR density (Fig 5) to allow an easier comparison between plots.
(TIF)

S4 Fig No of SAAR events and lengths distribution among Human orthologous proteins. For all the organisms under study, Human ortholog pairs with conserved SAARs were identified. For these proteins, the distribution of repeat length (X-axis) and number of events (Y-axis) are plotted.
(TIF)

S1 File Functional domain annotation for SAAR associated regions in human proteins. (XLSX)

S1 Table Codon and amino acid repeat length among orthologous genes associated with repeat expansion (CDS) diseases. (DOCX)
