# Supplementary figures and images for: Single Amino Acid Repeats in the Proteome World: Structural, Functional, and Evolutionary Insights
Source: PLoS One. 2016 Nov 28;11(11):e0166854. doi: 10.1371/journal.pone.0166854 (PMC5125637; doi:10.1371/journal.pone.0166854)

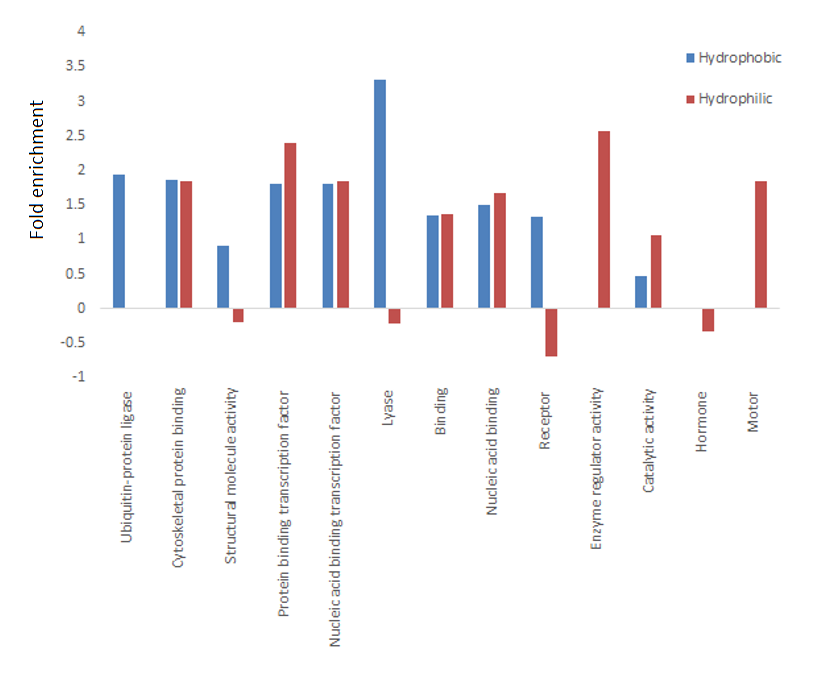

Supplement: S1 Fig — Distribution of various functional and molecular activity classes (X-axis) for SAAR associated proteins categorized based on their physical properties is shown. The functional classes were defined from Gene ontology annotations. The plot shows the fold enrichment (Y-axis) between expected and observed frequency in reference to the human proteome. The blue bars and red bars indicate hydrophobic and hydrophilic SAARs, respectively. (TIF) [file pone.0166854.s001.tif]

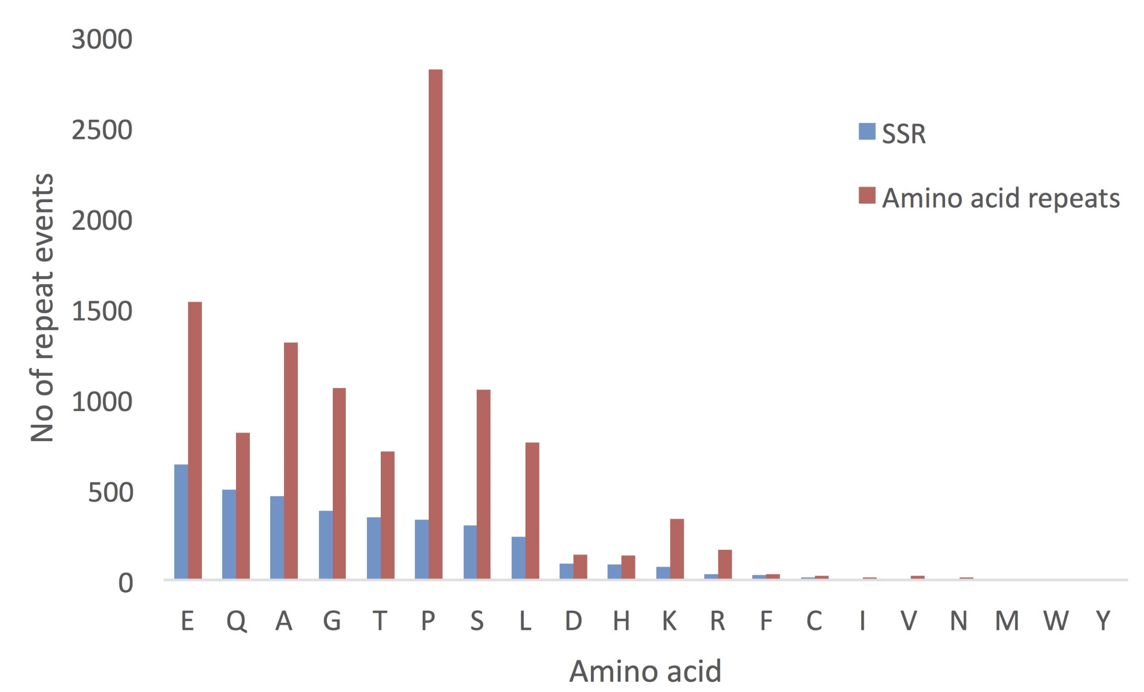

Supplement: S2 Fig — For all the coding regions corresponding to SAARs in the Human proteome, we calculated the number of times they were present as simple sequence repeats (SSRs). The X-axis shows the different amino acids and the Y-axis shows the number of repeat events in the proteome (red bars) and genome (blue bars). (TIF) [file pone.0166854.s002.tif]

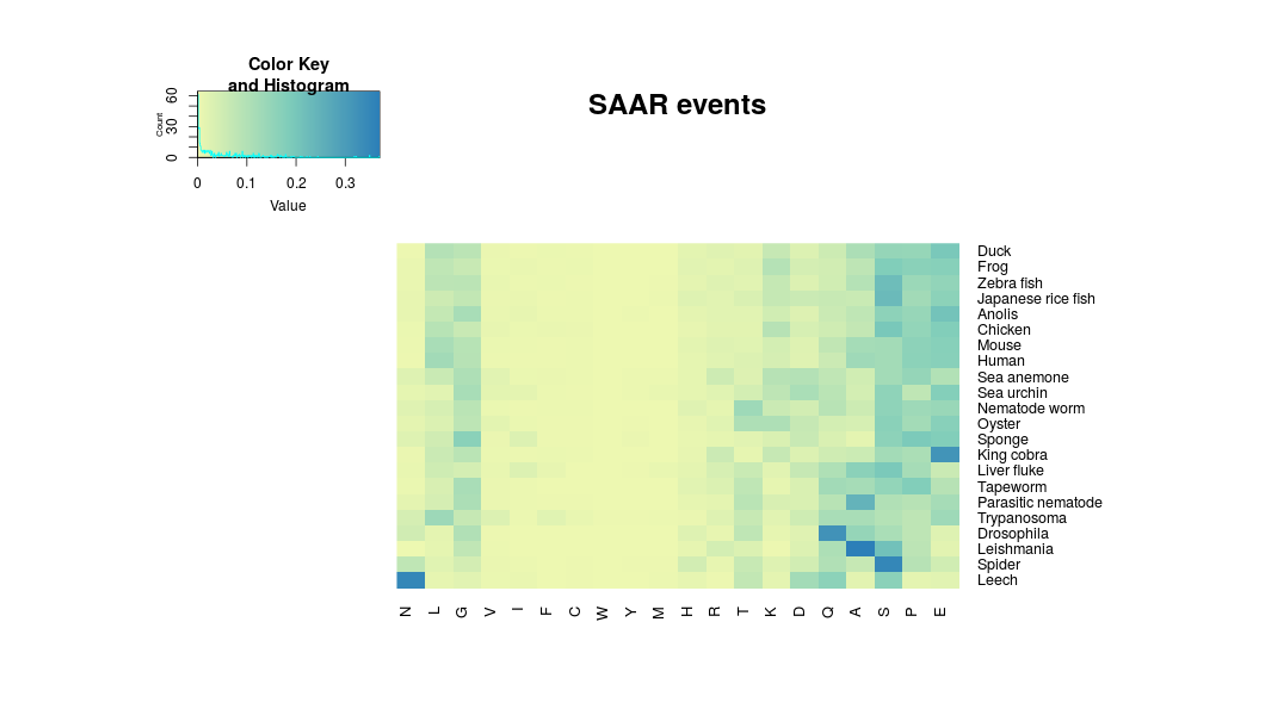

Supplement: S3 Fig — SAAR events were calculated and normalized to one million residues for the indicated proteomes and plotted as a heatmap where the X-axes show individual amino acid associated repeats and Y-axes have all the organisms under study. The order of amino acids and species was kept consistent with that of SAAR density (Fig 5) to allow an easier comparison between plots. (TIF) [file pone.0166854.s003.tif]

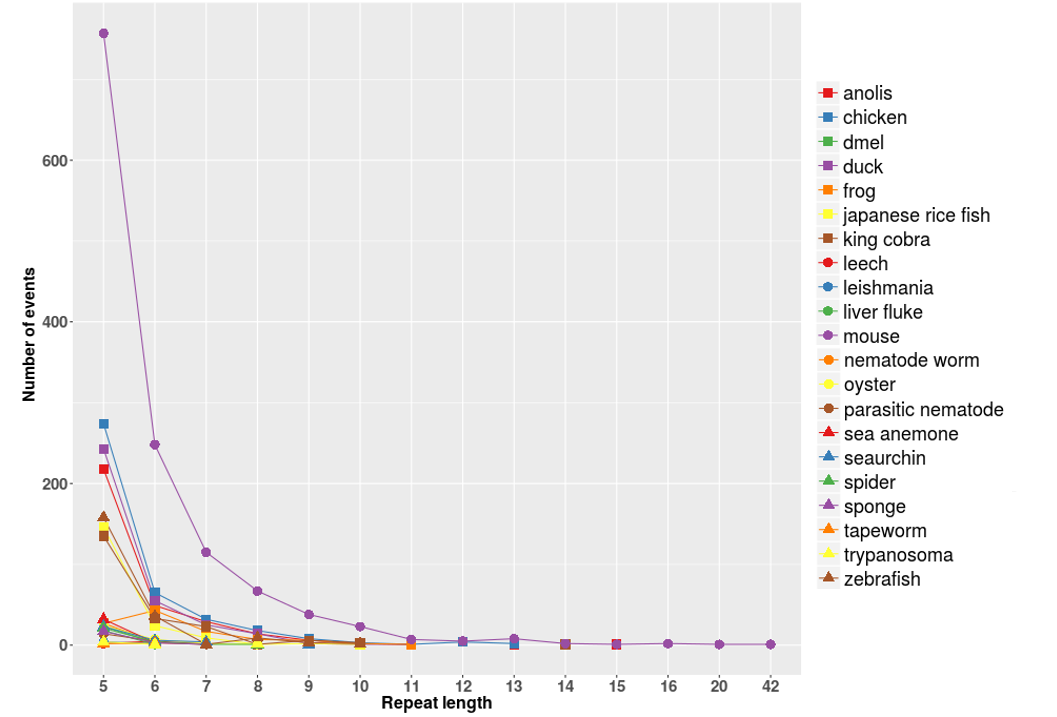

Supplement: S4 Fig — For all the organisms under study, Human ortholog pairs with conserved SAARs were identified. For these proteins, the distribution of repeat length (X-axis) and number of events (Y-axis) are plotted. (TIF) [file pone.0166854.s004.tif]
