# Supplementary material for: Single Amino Acid Repeats in the Proteome World: Structural, Functional, and Evolutionary Insights
Source: PLoS One. 2016 Nov 28;11(11):e0166854. doi: 10.1371/journal.pone.0166854 (PMC5125637; doi:10.1371/journal.pone.0166854)
Supplement: S1 Table — (DOCX) [file pone.0166854.s006.docx]

**S1 Table: Codon and amino acid repeat length among orthologous genes associated with repeat expansion (CDS) diseases**

|  | Huntingtin - HTT gene | | SCA3 - ATXN3 gene | | OPMD - PABN1 gene | |
| --- | --- | --- | --- | --- | --- | --- |
| Organism | CAG repeat length | PolyQ repeat length | CAG repeat length | PolyQ repeat length | GCG repeat length | PolyA repeat length |
| *Homo sapiens* | 19 | 23 | 8 | 10 | 6 | 10 |
| *Pan troglodytes* | 8 | 11 | 12 | 14 | 6 | 10 |
| *Macaca mulatta* |  |  | 7 | 7 |  |  |
| *Canis lupus familiaris* | 5 | 10 | 6 | 12 | 6 | 10 |
| *Bos taurus* | 15 | 15 | 0 | 0 | 6 | 10 |
| *Mus musculus* | 4 | 7 | 5 | 6 | 6 | 10 |
| *Rattus norvegicus* | 5 | 8 | 2 | 2 | 6 | 10 |
| *Gallus gallus* | 4 | 4 | 3 | 4 |  |  |
| *Xenopus (Silurana) tropicalis* | 3 | 4 | 2 | 2 |  |  |
| *Danio rerio* | 3 | 4 | 0 | 0 |  |  |
| *Caenorhabditis elegans* |  |  | 1 | 2 | 0 | 0 |
